# A Systems-Based Approach for the Prevention of Heat-Associated Kidney Disease in Latin American Workers

**DOI:** 10.5334/aogh.4760

**Published:** 2025-08-19

**Authors:** Miranda Dally, Lyndsay Krisher, Diana Jaramillo, Lee S. Newman, Joaquin Barnoya

**Affiliations:** 1 Climate Impacts on Worker Health & Safety Center, Colorado School of Public Health, University of Colorado, Anschutz Medical Campus, Aurora CO, USA; 2 Centers for Health, Work & Environment, Colorado School of Public Health, University of Colorado Anschutz Medical Campus, Aurora CO, USA; 3 Department of Environmental and Occupational Health, Colorado School of Public Health, University of Colorado Anshutz Medical Campus, Aurora CO, USA; 4 Department of Epidemiology, Colorado School of Public Health, University of Colorado Anschutz Medical Campus, Aurora CO, USA; 5 Division of Pulmonary Sciences and Critical Care Medicine, Department of Medicine, School of Medicine, University of Colorado Anschutz Medical Campus, Aurora, CO, USA; 6 Universidad Rafael Landivar, Guatemala City, Guatemala

**Keywords:** occupational health, kidney disease, heat-associated illness, implementation science

## Abstract

*Background:* The prevalence of chronic kidney disease and other non-communicable diseases in Guatemala has been rising. Recently, chronic kidney disease of non-traditional origin (CKDnt) has been identified among occupational cohorts and linked to heavy workloads in hot environments.

*Objective:* To describe a systems-based community participatory approach to occupational safety and health.

*Methods*: Over the last eight years, academic researchers have partnered with a large agribusiness in Southwest Guatemala to address the health, safety, and well-being concerns of workers, especially in relation to CKDnt. This case study presents the *Total Worker Health®* approach used to develop, implement, and evaluate interventions to address potential causes of CKDnt. The results of these interventions are presented using the Consolidated Framework for Implementation Research and RE-AIM.

*Findings:* A Total Worker Health (TWH) approach addressing both transactional and transformational organizational behavior change to reduce the incidence of reduced kidney function in this workforce can be successfully implemented. Our findings highlight the need for collaborative approaches to the development and implementation of strategies to adapt to climate change in the workplace. The sustainability of interventions needs constant re-evaluation to adapt to changing contexts.

*Discussion:* This case study illustrates a practical, evidence-based TWH approach to address the immediate impacts of heat stress and nephrotoxins on the kidney health of sugarcane workers in Guatemala. It also illustrates the importance of multi-modal interventions implemented and evaluated in a dissemination and implementation science framework informed by the community that is at risk. The principles underlying our approach may have applicability and generalizability to less extreme circumstances, and beyond those with heat-associated kidney disease.

## Background

While Guatemala is the largest economy in Central America, the poverty and income inequality rates among its 18 million people are among the highest in Latin America and the Caribbean [1]. The Pan American Health Organization (PAHO) estimates that the labor force (individuals aged 15–64) in Guatemala accounts for 63% of the population [2]. Nearly 1 in 3 Guatemalans works in agriculture [3], despite the limited opportunities for prosperity and growth in this work sector. Work is an important social determinant of health. While occupational safety and health statistics are, for the most part, unavailable for Guatemala, agricultural workers worldwide have among the highest risks for work-related injuries and illnesses. In addition to impacting income and financial security, “a person’s industry and/or occupation contribute to the burden and distribution of various chronic illnesses.” [4] Widely recognized among these illnesses is chronic kidney disease of non-traditional origin (CKDnt)—also known as Mesoamerican Nephropathy and Chronic Kidney Disease of unknown cause (CKDu).

It is increasingly understood that workers are the “climate canaries” [5], with little choice but to work under extreme climatic conditions, and suffer the health consequences [6]. Thus, this case study illustrates a systems-based, pragmatic, evidence-informed approach to the public health crisis facing the more than 2 billion workers globally currently exposed to conditions of extreme heat [7] and other climate hazards.

Since the 1970s, the prevalence of kidney disease (CKD) has been increasing in Central America [8]. The prevalences of CKD and end-stage kidney disease have been rising in Guatemala [9], especially in the South-Pacific agricultural regions [10–12]. While CKD is a well-known comorbidity of diabetes and hypertension, in the 1990s, clinicians in Latin America began to observe that young sugarcane workers were presenting with end-stage kidney disease unrelated to these common causes [13]. In 2002, Trabanino and colleagues published a landmark paper implicating work as a risk factor for CKDnt [14]. Over the past decade, we have documented a high incidence and prevalence of both acute kidney injury and CKDnt in Guatemalan sugarcane workers. We have also observed a suspicious excess of abnormal kidney function in young, first-time workers [15] consistent with other reports of community cases of CKDnt [16] and suggesting that both occupational and non-occupational community hazards may contribute to disease risk [13], although the prevalence and incidence of CKDnt in the general population remains unknown. Arroyo and coworkers recently reported an elevated prevalence in Guatemalan workers in melon, banana, and tomato production [17], and others in Latin America have reported disease in construction, brickmaking, and other non-agricultural industrial sectors [18, 19].

In exploring potential shared CKDnt disease risk factors in the workplace and community, researchers have pointed to the incessant rise in average ambient temperatures in the region as well as nephrotoxin and nephrotoxicant exposures such as pesticides, heavy metals, infections, and potentially nephrotoxic anti-inflammatory medicine use [13]. While the causes of CKDnt are likely to be multifactorial [13], occupational, and possibly community, heat exposure has been most frequently implicated as an important threat multiplier. The topography of Guatemala is rugged with elevations that go from sea level to 4,200 meters above sea level, resulting in six climate regions [20]. It is estimated that the average annual temperature in Guatemala increased by 0.5°C from 1990 to 2022 [20]. This increase is particularly troublesome for agricultural workers who labor long hours at high levels of exertion outdoors [7] making them more susceptible to climate-related changes in heat than some other workforces.

Climate factors such as heat extremes can act synergistically with occupational and non-occupational-related exposures such as air pollution and social determinants of health to exacerbate pre-existing diseases and vulnerabilities, such as cardiovascular and respiratory disease risk [21]. In the instance of climate-associated CKDnt, and based on the line of evidence implicating work- and non-work-related factors in its pathogenesis [13], we have successfully pursued a more holistic approach to developing preventive strategies both at home and work using the framework referred to as *Total Worker Health®*. Developed in 2011 by the U.S. Centers for Disease Control and Prevention’s National Institute for Occupational Safety and Health (NIOSH), Total Worker Health (TWH) integrates protection from work-related safety and health hazards with the promotion of injury and illness-prevention efforts to advance worker well-being, addressing a range of health conditions including not only occupational injuries, but chronic diseases as well [22].

## Approach

Grupo Pantaleon (Pantaleon), a Guatemalan agro-industrial company, partnered with the University of Colorado’s Centers for Health, Work & Environment (CHWE) to address rising kidney disease rates in its workforce. This case study examines the collaborative approach to preventing CKDnt, a health condition amplified by heat and humidity exposure during labor. Pantaleon aimed to improve the well-being of its employees by implementing more effective mitigation strategies in response to climate pressures. This case study examines the implementation process and outcomes of these strategies, and the overall efficacy in addressing CKDnt in the workplace.

### Consolidated framework for implementation research

To evaluate the implementation process and identify successful strategies to address CKDnt in Pantaleon’s workforce, we used a modified Consolidated Framework for Implementation Research (CFIR) [23]. CFIR has five major domains: (1) innovation, or the *what* that is being implemented, (2) the outer setting and (3) the inner setting, or the *where* the intervention is being implemented, (4) the individuals, or the *who* is involved in the implementation, and (5) implementation process, or the *how* it is being implemented.

The CFIR was chosen because, as Guerin and colleagues note, understanding which implementation strategies (or bundles of strategies) are most appropriate is context dependent [24]. The outer and inner setting domains of CFIR allow us a structured way to describe these contexts. Especially important for understanding the context in which TWH interventions are implemented is the inner domain, which allows us to understand the workplace culture in which TWH interventions are being tested.

**Figure 1:**
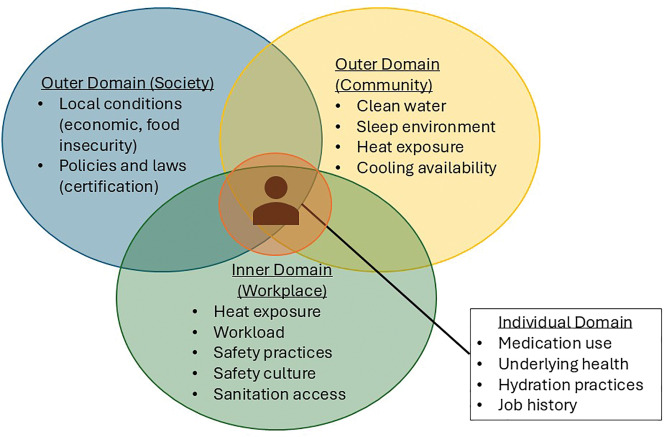
Framework for developing heat-associated CKDnt interventions for the workplace using the Total Worker Health approach.

*Innovation*. The TWH framework allowed us to identify the constructs that influenced the development of CKDnt and that drove our selection of interventions. Under the TWH framework, we assumed that the development of CKDnt was not solely through occupational heat exposure, but rather the interaction of occupational heat exposure with individual, community, and societal vulnerabilities. We postulated that *in addition to* occupational conditions: (1) individual factors influence an individual’s heat tolerance; (2) community/household-level factors, such as access to clean water, cooking practices, cooling options, and sleep environments conducive to physical recovery; and (3) economic burden, access to healthcare, and food insecurity may amplify the risk of CKDnt among heat-exposed workers Figure 1. To address these factors, we developed pragmatic TWH pilot interventions aimed at prevention strategies such as hydration, rest, shift length, and schedule, for groups of workers to improve cross-work shift as well as pre- and post-harvest kidney function, muscle breakdown, and other climate-associated health effects. This resulted in a total of 7 interventions that are described further in Table 2.

In the broadest terms, we approached intervention development and selection through the lens of organizational behavior change [25]. Our shared strategy was twofold. First, we sought to take a “transactional” approach to our interventions [25–27]. The transactional approach aims to “fix immediate problems” through addressing the structure of work (e.g., matching task requirements with individual skills/abilities), management practices, and systems (e.g., policies and procedures) [25]. We applied community-based participatory research [28] principles to address potential causes of CKDnt. Second, over an extended time span, we promoted organizational culture change, so-called “transformational” change, with the goal of improving company leadership understanding, adoption, and maintenance of TWH principles for worker health and safety as core corporate values. This transformation approach takes a long-range view toward improving organizational culture with the goal of improving the likelihood of long-term adoption, adaptation, and maintenance of TWH interventions. Further details on each intervention and reason for selection are provided in Table 2.

*Outer domain.* At the national level, each country is influenced by cultural values, political and economic structures, and available community resources [29]. As discussed above, the broader epidemic of CKDnt was being recognized throughout the region, resulting in increased pressure to identify risk factors and to intervene. To address this, the Consortium for the Epidemic of Nephropathy in Central America (CENCAM) was developed as a way for academic researchers, policymakers, environmental and labor organizations to share findings and recommendations for best practices.

*Inner domain.* Cultural values shape senior leadership attitudes toward the development of TWH policies and programs and their implementation in practice as manifested by a culture of TWH in the entire organization [29]. Our interventions were implemented in an organization whose organizational culture emphasized productivity while prioritizing workforce health and safety. The company’s mission is to lead the industry in workplace health and safety, and active collaboration with academic partners facilitates the adoption of evidence-based strategies, ensuring that resources are used effectively to support both productivity and employee well-being.

*Individual domain*. Implementation required collaboration at multiple levels within Pantaleon and between Pantaleon and CHWE (Table 1). CHWE investigators worked side-by-side with the agribusiness’s clinical, environmental health and safety, and social responsibility staff to prioritize health and safety concerns and develop intervention protocols. Pantaleon staff drove field implementation, with periodic consultation with CHWE. When Pantaleon implemented a new program, policy, or practice change based on recommendations, CHWE provided support to clinical, human resources, and managers who developed implementation protocols for the new strategies and helped with capacity building by training health and safety staff in research principles and practice. CHWE support included independent audits of the implementation of the intervention, evaluation of data, and providing research support staff to help conduct the intervention when necessary.

**Table 1:** Individuals and roles involved in the delivery of TWH interventions to address CKDnt in the workplace.

INDIVIDUALS	ROLE
Corporate leadership (sustainability, HR, C-level)	Decision-making Resource allocation Establishing health and safety as a corporate priority Communications Logistical support
Local leadership (on-site company leadership, occupational safety and health)	Decision-making, implementation leads, innovation deliverers, Interpretation of results
Centers for Health, Work & Environment researchers	Research design leadership Innovation deliverers Logistical support Implementation team members Collaboration building Workforce capacity building Evaluation and identification of evidence base Data analysis and interpretation Communication of results to partners, leadership, stakeholders
Local research staff	Other implementation support
Field managers (field worker supervisors)	Innovation deliverers, problem-solving Local communications Integration of health and safety priority into work processes
Local medical staff	Innovation deliverers Communication with workers Research design contributors Implementation deliverers
Field workers (sugarcane harvesters)	Innovation recipients Contributors to design elements (e.g., choices of personal protective equipment, types of electrolyte solution)

*Implementation Process*. Our pragmatic TWH pilot interventions applied principles rooted in dissemination and implementation science, addressing a gap in the field of CKDnt intervention research [24, 30]. While a few workplace intervention studies exist to address CKDnt [31–33], to our knowledge, none of these studies have taken a dissemination and implementation science approach when presenting their effectiveness findings [34, 35]. However, our experience, and that of NIOSH, supports the need to be comprehensive in both the approach and evaluation of workplace-based interventions [36].

The process started in 2016 when members of Pantaleon’s leadership and occupational safety and health team joined together with researchers from CHWE. The first step in the partnership was for CHWE to assess the needs of Pantaleon and its stakeholders as well as understand what steps had already been taken to try to address the problem. This community-based participatory approach [37] to the needs assessment included surveying workers on demographics, work practices and injuries, and health/health risk behaviors, as well as working with Pantaleon leadership to identify priority areas of concern. Annual scopes of work were jointly developed to outline the annual project goals, milestones, and roles and responsibilities [23]. Participation in the interventions was encouraged by on-site medical staff, a trusted source of information among workers.

**Figure 2:**
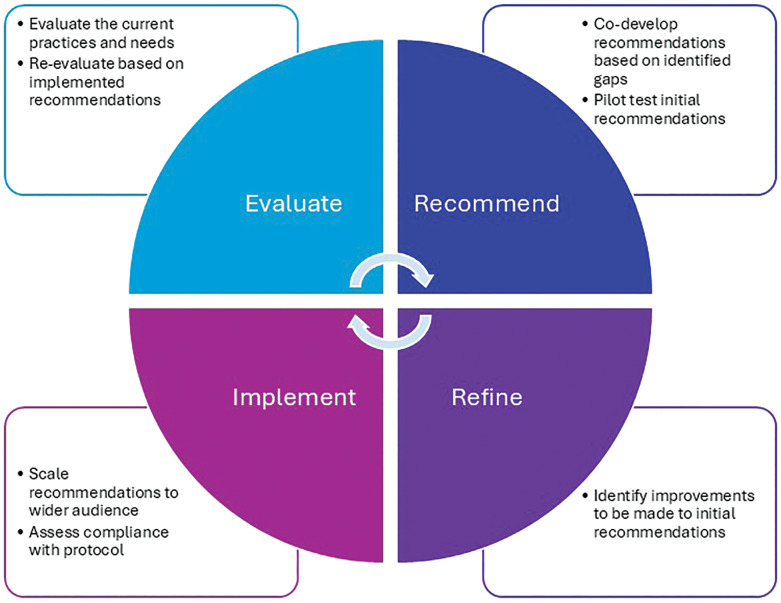
Approach to continual CKDnt intervention improvement.

Through a cyclical process, we were continuously evaluating, recommending, refining, and implementing (Figure 2). Recommendations to the agribusiness for new protective strategies and interventions were developed in partnership with Pantaleon to help ensure that they were adoptable and sustainable if they showed promise of efficacy. Scalability of the recommendations was also a major consideration, knowing that well-intentioned and efficacious pilot studies do not necessarily succeed when scaled up from several hundred workers to thousands. Evaluation of the impact of these strategies incorporated feedback from workers, supervisors, and leadership in addition to our research findings (evaluation of the effectiveness of the pilots). As we have noted previously, “the practice of continuous evaluation and quality improvement fit within Pantaleon’s commitment to building and maintaining a strong culture of safety, health, and wellbeing that encourages and promotes active participation among employees [38].” Notably, we have identified scalability to be a staged process which requires monitoring to address when processes and procedures are not being adhered to and may lead to retraining and re-educating those responsible for implementation. Leadership and supervisor turnover required periodic meetings to educate and reinforce the value of the interventions and ensure support for scaling.

### Evaluation of implementation outcomes

To assess the implementation outcomes of each of the individual TWH interventions, we applied the RE-AIM framework. RE-AIM is a framework that can be used to evaluate the internal validity of effective interventions when applied in practice [39]. Internal validity is especially important when translating interventions into the workplace, given the potential influence of inner domains on efficacy. For each of the 7 interventions presented in this case study we evaluated how many workers participated in the intervention (reach), the fidelity of the intervention (implementation), the impact of the intervention on the target outcome (effectiveness), whether the intervention has been integrated into current practice (adoption), and if and how the intervention is operationalized currently (maintenance). A summary of the RE-AIM measures for each of the interventions is provided in Table 2.

**Table 2:** Summary and results of TWH interventions to address CKDnt prevention strategies focused on the individual, workplace, community, and societal domains.

TWH INTERVENTION^*^ (HARVEST SEASON)	DESCRIPTION/INTERVENTION DESIGN (TWH DOMAIN)	INTERVENTION SELECTION	IMPLEMENTATION OUTCOMES
Worker heat stress education program (2016–2017)	Augmented current worker education program to include the importance of hydration, rest, and shade and the need to moderate their use of NSAID. Trained Pantaleon nurses to provide education through face-to-face communication, posters, and pocket urine color charts for hydration status self-evaluation in Spanish and adapted to low literacy. (Individual)	Evidence Base: OSHA Water, Rest and Shade program Relative Advantage: Workers and supervisors have knowledge of the importance of hydration and shaded rest breaks. Adaptability: Educational materials were adapted for low literacy. Trialability: Straightforward Complexity: Straightforward Cost: Minimal; printing costs and time for education delivery (< 5 minutes) Organizational behavior change: Transactional	Reach: 517 sugarcane workers participated in the initial study. Implementation: Delivery of the intervention was driven by field nurses, as it was integrated within their existing educational program. Therefore, the approach was designed to be feasible and culturally acceptable in this setting from the outset. Effectiveness: Workers demonstrated greater understanding of the signs and symptoms of dehydration [40]. Adoption: The program was rolled out company-wide to also include workers inside the processing mill and in other heat-exposed, non-field operations. Maintenance: Health and safety personnel at the company conduct random urine checks with workers throughout the harvest. The heat stress education program continues to be an integral component of the education program delivered daily by the nurses.
Wellness Incentive (2016–2017)	Workers who started the shift hydrated (urinary specific gravity was ≤ 1.020) or who maintained or improved their hydration across the shift (< 1% body weight loss) were entered into a raffle to win prizes (e.g., soap, socks, soccer balls). Those not considered hydrated received additional education to drink more water and take more rest. (Individual)	Evidence Base: Incentives are an effective way of affecting healthy behavior in workplace settings. Relative Advantage: Promotes hydration among workers, both on and off-work Adaptability: Ability to adjust to the number of workers and their needs of water and electrolyte consumption. The incentive raffle is a useful employee engagement tool that was later adapted for use in other projects in this setting given its success here. Trialability: Was carried out with 10 working groups Complexity: Straightforward Design: Cross-sectional and longitudinal Cost: Costs minimal; dependent on local pricing of incentives Organizational behavior change: Transactional	Reach: 517 workers Implementation: The raffle was popular and well-accepted by participants Effectiveness: The percentage of workers who were dehydrated at the beginning of the workday was reduced [41]. The percentage of those dehydrated at the end of the shift was already low (2%) and remained unchanged. Adoption: Workers were consuming too much plain water and not enough electrolytes, indicating a need to refine the intervention [42]. Maintenance: The incentives were not continued after the intervention, but the company continues to invest resources in hydration programs.
Electrolyte Study (2017 –2018)	A three-week study that progressively increased the recommended volume of electrolyte solution for workers to consume. Pantaleon was responsible for providing workers with the recommended volume of electrolyte solution. (Individual, Workplace)	Evidence base: NIOSH recommendations Relative advantage: Workers were able to replace needed electrolytes lost in sweat. Adaptability: WHO rehydration solution formula was followed and produced locally for the intervention. Trialability: Was able to be piloted with a single work group Complexity: Straightforward Design: Longitudinal (3 weeks including baseline) Costs: Laboratory costs associated with health monitoring (blood and urine), pre- and post-intervention, survey development	Reach: 50 of the workers participated in the intervention (1 complete work group/turma) Implementation: Dislike of certain flavorings expressed among workers Effectiveness: When workers increased electrolyte solution consumption, they experienced less muscle damage and less self-reported weakness [43]. Kidney function was maintained. Serum electrolyte balance achieved. Adoption: The electrolyte solution was provided to workers in powder form to facilitate widespread distribution of larger quantities of electrolytes across the company’s farms (previously the solution had been pre-mixed in 500 ml pouches) Maintenance: Continuous evaluation is needed to ensure access to the recommended quantities of electrolytes, and that expected health effects are maintained.
6-Hour Workday Study (2017–2018)	A three-week study that reduced the number of hours a group of harvesters worked. Daily hours were reduced from 8 to 6. Supervisors at Pantaleon were responsible for ensuring the recommended schedule was adhered to. (Workplace)	Evidence Base: OSHA recommendations [44] Relative Advantage: Reduces the number of hours workers are exposed to heat Adaptability: Ability to adjust the number of reduced hours to meet the needs of the company. Trialability: Was able to be piloted with a single work group Complexity: Straightforward Design: Comparative effectiveness trial Cost: Laboratory costs associated with health monitoring (blood and urine), pre- and post-intervention, survey development; may result in reduced productivity. Organizational behavior change: Transactional	Reach: 50 of the workers participated in the intervention (92% of eligible, 1.6% of the total workforce) Implementation: Potential issues with adherence to the recommended schedules. Effectiveness: No observed effect on post-shift measurements of kidney function or incidence of acute kidney injury. Less muscle breakdown was observed during the weeks with the shortened shift. Workers on average produced less sugarcane during the shorter shifts. Adoption: The pilot intervention was trialed in one of 49 workgroups (2%). Maintenance: Was not continued after pilot. Pantaleon is currently considering schedule refinements and re-evaluation of reduced schedules and/or earlier shift start times to avoid the hottest times of day.
Mid-Harvest Season Screening (2017–2018)	Workers’ kidney function was evaluated mid-way through the harvest to identify: (1) abnormal kidney function group, (2) reduced kidney function group, and (3) normal kidney function group. Those with reduced or abnormal kidney function were referred to the occupational medicine physician employed by Pantaleon. Based on the severity of the abnormal kidney function reading, workers either received consultation and one-on-one education and were re-tested 2 weeks later, or they were referred to the local nephrologist or hospital and removed from work. The company continued to pay their wages for the rest of the season. Those with stable or improving kidney function continued to work in jobs with lower physical demands and lower heat exposure (Workplace, Society).	Evidence Base: Occupational health surveillance Relative Advantage: Identifies health conditions closer to onset to identify preclinical decline of kidney function and allow for work modification and clinical care for affected workers Adaptability: Can modify the timing of screening Trialability: Dependent on the incidence of disease Complexity: Straightforward Design: Cross-sectional Cost: Equipment costs for point-of-care testing, personnel to conduct screenings and review results. Costs increase with severity and number of cases identified. Potential impact on job assignments and productivity Organizational behavior change: Transactional	Reach: 483 of 517 eligible workers Implementation: Some workers were not present on days when screening occurred. Effectiveness: Over three quarters of those with abnormal or reduced kidney function at mid-season had kidney functions that improved by the end of the harvest. Those with early indications of kidney function decline received early clinical attention and modification of work duties [45]. Adoption: The company developed a protocol for mid-harvest screening. Maintenance: Continuous evaluation to ensure protocol is adhered to.
TWH Leadership Training (2019–2020)	Health, safety, and well-being culture survey was distributed to a representative sample of workers across the workforce. Ten TWH leadership trainings were provided across three countries (four sites) of Pantaleon operations, reaching 121 leaders. A study of 1,861 workers showed that field workers with physically demanding jobs and administrative workers both reported more work-related stress. (Workplace)	Evidence Base: SSWell Study [46, 47] Complexity: Moderate Design: Cross-sectional Cost: Company contracted with CHWE to collect and analyze health and safety data, create training materials, and deliver trainings. Costs to the company include allocating work time for occupational safety and health staff to help co-develop the training, allocating work time for leaders to participate in pre-training interviews and surveys, attending 8- hour in-person training, completing follow-up surveys. Advantage: Reached leadership at all levels and provided leadership training including feedback from employees and top management Adaptability: Ability to adjust the training to all leadership levels. Training was also adapted to the needs of the company and diverse countries. Trialability: Can be tested in individual work units Organizational behavior change: Transformational	Reach: A total of 120 leaders participated in the Total Worker Health (TWH) training, informed by a Needs Assessment. We surveyed a 20% convenience sample from each department, except Agriculture, where we sampled 297 workers (3% of the 9,744 Agriculture employees), totaling 1,541 surveys [48, 49]. Implementation: Some challenges with follow-up were identified, which may affect sustainability. Effectiveness: Leaders reported improved leadership practices immediately post-training, sustained during the 3-month follow-up. Health indicators showed reduced stress and improved well-being. High job demands were linked to poorer health among field and administrative workers, prompting recommendations for adjustments. [48, 49] Adoption: The training was delivered at one timepoint, with a 3-month follow-up. Maintenance: Pantaleon continues to focus on the key areas identified in the training and has trained clinical staff through additional national and international programs. Principles taught in these sessions have been incorporated into the formal training received during the employee onboarding process for all new and returning Pantaleon workers.

*Ethical approvals for these projects were obtained from the Colorado Multiple Institutional Review Board (COMIRB). Informed consent was obtained from study participants for all studies considered as human subjects research. Studies deemed human subjects research also received approval in Guatemala from either the Comite de Etica, Facultad de Medicina, Universidad Francisco Marroquin-Hospital Universitario Esperanza or ZUGUEME Comité Ética Independiente.

## Findings

Table 2 provides a summary of the findings from each individual intervention. Our overall results cannot be attributed to any single intervention. Rather, these results demonstrate how addressing an occupational health concern using a TWH framework and dissemination and implementation sciences approach can improve worker health outcomes in the face of extreme meteorologic conditions and in the context of high metabolic load and potentially multiple environmental co-exposures. It also illustrates the need for an agile response to changing conditions and changing results of evaluations.

### Additional total worker health evaluations

In addition to the TWH interventions, observational studies were conducted to assess non-strictly workplace-based factors, including nutrition and post-work recovery. A nutritional assessment of the current daily meal plan provided to the workers was developed by CHWE and conducted by a team of faculty and students from the Universidad Francisco Marroquin in the 2018–2019 harvest season. Nearly half of the study participants lost weight, fat, and muscle mass (i.e., non-fat mass) during the season. Anemia was present among 13% of workers [50]. Recommendations were made to modify the current nutrition program. Pantaleon continues to conduct empiric studies to promote better nutrition.

In the 2022–2023 harvest season, the CHWE team collected environmental and internal core body temperature data from workers, continuously at work and during the off-work/overnight period. Workers were surveyed about their recovery practices after work. Team members, with permission, conducted assessments of the home. Workers experienced high rates of heat strain during the workday. Core body temperatures continued to rise during the workday, and for some, after work as well. Preliminary data showed elevated temperatures at workers’ homes. Workers’ home environments and available resources may not be conducive to recovery from heavy labor in the heat [51]. Pantaleon leadership has been briefed on these findings and is currently considering how to contribute to off-work recovery from the day’s labor in extreme heat. This illustrates the ongoing, iterative, continuous improvement process.

### Efficacy and effectiveness

The primary goal of this case study was to take a systematic approach in describing the development and implementation of TWH interventions to address CKDnt in the workplace. While not intended to evaluate the effectiveness of these programs, we have noted a decrease in the incidence of reduced kidney function over the course of this project. In the 2015–2016 harvest year, we estimated that 10% of workers finished the harvest with reduced kidney function, defined as eGFR < 90 mL/min/m^2^ [52]. In the 2021–2022 and 2022–2023 harvest years, it is estimated that this number decreased to 7% of workers (under review, see study details in Dally et al., 2025 and Adgate et al., 2025) [53, 54]. We acknowledge many external factors may have contributed to this observed reduction in incidence and cannot attribute these results solely to the TWH interventions. We have observed that incidence continues to fluctuate from season to season, highlighting that external factors such as climate and environment may influence efficacy, and there is a need to remain vigilant in evaluating and modifying interventions. This observation reinforces another important consideration for any set of interventions, namely, the need for ongoing monitoring and auditing to ensure that when best practices are implemented, they continue to be adhered to, and demonstrate ongoing effectiveness in dynamic, real-world settings.

## Discussion

Across the globe, workers face increasingly challenging conditions because of the climate’s accelerating effects on their health, safety, and productivity. Climate factors, such as increasing heat, are threat multipliers, compounding the effects of traditional workplace hazards on the more than 2.4 billion workers exposed to heat extremes each year. Workers are vulnerable bellwethers of a crisis that affects them now and that will impact us all unless we develop more effective and sustainable solutions. Workplaces, especially in the tropical and subtropical zones of Lower Middle-Income Countries (LMIC), may hold the key to solutions that mitigate climate harm worldwide. This case study illustrates a practical, evidence-based TWH approach to address the immediate impacts of heat stress and nephrotoxins on the kidney health of sugarcane workers in Guatemala. It also illustrates the importance of multi-modal interventions implemented and evaluated in a dissemination and implementation science framework informed by the community that is at risk. The principles underlying our approach may have applicability and generalizability to less extreme circumstances, and beyond those with heat-associated kidney disease. We draw several lessons from this case study.

**Solutions are possible but require both community engagement and tending.** To mitigate harm to workers, we relied on evidence and the experience of people working in the fields to adapt “high level” general recommendations (like “water, rest, shade”) to local conditions. Adoption of so-called best practices required adaptation based on the collective input of those affected, which in this case included the employer and workers. Both initial and sustained benefit could only be achieved by having the buy-in of company leadership, supervisors, and workers. Stakeholders benefited from immediate, evident results. Even so, periodic evaluation of the implementation process itself, of the resulting changes in working conditions, and of data on health outcomes was needed to monitor for adoption drift and sustain even simple changes in how people approached their work and heat mitigation. We found that the rate of adoption of new protective measures was helped by sharing data based on measurement of short-term/immediate changes in the heat mitigation process (e.g., access to and consumption of water and electrolytes) and in same-day measures of health effects (e.g. fatigue, electrolyte balance, muscle injury, and kidney function).

**The solutions to heat-related illness in the workplace require a systems approach to change organizational behavior and improve culture.** In this case study, we applied principles of organizational behavior change. We aimed to achieve tactical (“transactional”) changes in the organization to show employees and managers that near-term improvements in working conditions were occurring and beneficial. At the same time, because of a highly engaged group of corporate leaders, we collaborated on a long-term culture change process: to incorporate worker health and well-being into the organization’s core values (so-called “transformational” change). The latter involved leadership training, communication of core principles throughout the organization, and demonstrated ongoing commitments to worker well-being and illness prevention as quality improvement goals. This approach has positioned the company to identify and respond to other endemic and emerging threats to worker well-being and productivity. This has been illustrated by their proactive response to the COVID-19 pandemic which prioritized worker safety, immunization, and medical care, and their development of a hearing conservation and noise mitigation program to prevent occupational hearing loss in mill workers [55, 56]. **Recommendation:** Identify, implement, and test practical solutions to achieve near-term “wins.” Work with decision makers so that they begin to transform their culture. Focus on leadership that communicates the significance of climate impacts on their business and workforce, values investment in climate mitigation solutions, and prepares for a sustained effort in adapting to changing conditions.

**Total Worker Health® is a useful framework for addressing heat-associated illness, because part of the solution is at work, but off-work factors must be taken into account to find a solution.** As discussed above, NIOSH has promoted an integrated approach to developing policies, practices, and programs to improve worker health, safety, and well-being. We found that this approach resonates with employers who are tackling heat-associated illness: responsibility for the health of workers is not solely the responsibility of the individual. In this case study, Pantaleon leadership already recognized that there were actions to be taken to prevent occupational injuries and create a safety culture. We benefited from prior efforts at fostering a safety culture, building on that transformational effort to take a more human-centered approach to business operations. Pantaleon was early in recognizing that heat stress raises those risks, especially kidney injury. But moreover, through corporate leadership training on TWH principles [29, 48, 49], company managers began to identify how they can help promote worker health and well-being. Through an ongoing evaluation program with Pantaleon, we have now determined that additional organizational changes are needed that will take into consideration workers’ living conditions and health behaviors. The priority is to mitigate heat stress on the job, but upcoming approaches will increasingly consider the off-work period when many workers may be unable to escape the heat due to poor living conditions. A Total Worker Health mindset opens the door to this broader approach. For example, as mentioned in Figure 1, broader, industry-wide and society-wide change is still needed to mitigate financial toxicity that motivates workers to perform high physical exertion. There is a need for industry and societal reforms that guarantee workers a fair and just living wage and that do not rely on pay-for-performance incentives that exacerbate heat-associated illness. In the absence of country-wide wage reform, company-level efforts to counter-balance productivity incentives—like the wellness incentives provided by many companies in high-income countries we piloted during the 2016–2017 harvest—may help. **Recommendation:** Work with business leaders and stakeholders to improve their understanding that the policies and practices of their organization can have both positive and negative effects on the health of workers exposed to climate hazards.

**Our approach to mitigating heat stress applies principles grounded in dissemination and implementation science.** In this case study, the design and evaluation process followed the RE-AIM framework [24,30], one of the many valid methodologies now being applied to a wide range of occupational and public health challenges. The objective has been to co-develop solutions to mitigating heat stress and exposures to nephrotoxins by considering each intervention element’s Reach, Efficacy/Effectiveness, Adoption, Implementation, and Maintenance (sustainability). Taking this approach to primary prevention allowed us to understand the local conditions and to tailor recommended best practices so that they were culturally relevant. We also demonstrated how ongoing medical screening can be used not only as a primary but also as a secondary prevention [45]. People found to be in the early stages of kidney dysfunction were referred for medical care and placed in lighter duty jobs, resulting in improvement or stabilization of their kidney injury. Notably, no single strategy was implemented in a silo, but rather synergistically with one another. We learned that Pantaleon, like other businesses, has a different vocabulary but similar approaches to the design, implementation, review, and improvement of production processes and that they can be applied to finding solutions to heat-associated illnesses. **Recommendation:** Encourage organizational behavior change by having businesses apply the same operating principles that they use to run a successful business to how they implement and operate climate-related health, safety, and well-being solutions.

**Partnering with Industry Leaders can help scale out climate interventions for greater impact.** Our previous research in the US demonstrated that it is possible to leverage the implementation science framework to scale out TWH interventions to employers across a range of industries [57]. In addition to showing applicability to a single company in an LMIC in Latin America, we have observed that the successes at Pantaleon resulted in changes in kidney disease prevention practices by the other major sugar producers in Guatemala, who are members of a guild association. Organizations such as the Association of Sugar Producers of Guatemala (ASAZGUA) play a role in promoting best practices across an industry. In this example, they support social programs aligned with the Sustainable Development Goals promoted by the United Nations and a Climate Change Institute to understand and mitigate climate impacts [58]. A broad public health impact is within reach through public–private partnerships [38], including at the highest levels of government, including the G20 nations who participate in the United Nations’ International Labour Organization’s Vision Zero Fund efforts to address the impact of climate change and heat on workers across the international supply chain [7]. **Recommendation:** We encourage occupational health providers and climate and health scientists (in high- and LMICs) to partner with like-minded employers to mitigate climate change. Those organizations become thought leaders in their communities, extending the impact of research that is put into practice. The diffusion of innovation at a business-to-business level can have positive impacts on those with individual- and community-level vulnerabilities when organizations model-the-way and advocate for equitable workplace-level policies and practices [59].

## References

[r1] The World Bank. The World Bank in Guatemala. 2024. Accessed July 1, 2024. https://www.worldbank.org/en/country/guatemala/overview.

[r2] Pan American Health Organization. Country Profile Guatemala. Published October 2024. Accessed July 1, 2024. https://hia.paho.org/en/countries-22/guatemala-country-profile.

[r3] USAID. Food Security. Accessed July 1, 2024. https://www.usaid.gov/guatemala/our-work/food-security.

[r4] Armenti K, Sweeney MH, Lingwall C, Yang L. Work: A social determinant of health worth capturing. Int J Environ Res Public Health. 2023;20(2):1199. 36673956 10.3390/ijerph20021199 PMC9859245

[r5] Roelofs C, Wegman D. Workers: The climate canaries. Am J Public Health. 2014;104(10):1799–1801. 25122009 10.2105/AJPH.2014.302145 PMC4167120

[r6] Schulte PA, Jacklitsch BL, Bhattacharya A, et al. Updated assessment of occupational safety and health hazards of climate change. J Occup Environ Hyg. 2023;20(5–6):183–206. 37104117 10.1080/15459624.2023.2205468 PMC10443088

[r7] International Labour Organization. Ensuring Safety and Health at Work in a Changing Climate. Geneva: International Labour Office; 2024.

[r8] Wesseling C, van Wendel de Joode B, Crowe J, et al. Mesoamerican nephropathy: Geographical distribution and time trends of chronic kidney disease mortality between 1970 and 2012 in Costa Rica. Occup Environ Med. 2015;72(10):714–721. 26199395 10.1136/oemed-2014-102799

[r9] GBD 2017 Disease and Injury Incidence and Prevalence Collaborators. Global, regional, and national incidence, prevalence, and years lived with disability for 354 diseases and injuries for 195 countries and territories, 1990–2017: A systematic analysis for the Global Burden of Disease Study 2017. Lancet. 2017;392(10159):1789–1858. 10.1016/S0140-6736(18)32279-7 PMC6227754 30496104

[r10] Garcia P, Mendoza C, Barnoya J, et al. CKD care and research in Guatemala: Overview and meeting report. Kidney Int Rep. 2020;5(9):1567–1575. 32954082 10.1016/j.ekir.2020.06.020 PMC7486195

[r11] Laux TS, Barnoya J, Cipriano E, et al. Prevalence of chronic kidney disease of non-traditional causes in patients on hemodialysis in southwest Guatemala. Rev Panam Salud Publica. 2016;39(4):186–193. 27657183

[r12] Laux TS, Barnoya J, Guerrero DR, Rothstein M. Dialysis enrollment patterns in Guatemala: Evidence of the chronic kidney disease of non-traditional causes epidemic in Mesoamerica. BMC Nephrol. 2015;16:54. 25881146 10.1186/s12882-015-0049-x PMC4406024

[r13] Johnson RJ, Wesseling C, Newman LS. Chronic kidney disease of unknown cause in agricultural communities. N Engl J Med. 2019;380(19):1843–1852. 31067373 10.1056/NEJMra1813869

[r14] Trabanino RG, Silva CR, Mercado MO, Merino RL. End-stage renal disease among patients in a referral hospital in El Salvador. Rev Panam Salud Publica. 2002;12(3):202–206. 12396639 10.1590/s1020-49892002000900009

[r15] Dally M, Butler-Dawson J, Cruz A, et al. Longitudinal trends in renal function among first time sugarcane harvesters in Guatemala. PLoS One. 2020;15(3):e0229413. 32142520 10.1371/journal.pone.0229413 PMC7059928

[r16] Ceron A, Ramay BM, Mendez-Alburez LP, Lou-Meda R. Factors associated with chronic kidney disease of non-traditional causes among children in Guatemala. Rev Panam Salud Publica. 2021;45:e24. 33727908 10.26633/RPSP.2021.24 PMC7954194

[r17] Arroyo G, Soto G, García S, et al. Prevalence of kidney disease of unknown etiology in agricultural workers, Guatemala. Rev Panam Salud Publica. 2023;47:84. 10.26633/RPSP.2023.84 PMC10231270 37266488

[r18] Gallo-Ruiz L, Sennett CM, Sanchez-Delgado M, et al. Prevalence and risk factors for CKD among brickmaking workers in La Paz Centro, Nicaragua. Am J Kidney Dis. 2019;74(2):239–247. 30826087 10.1053/j.ajkd.2019.01.017

[r19] Keogh SA, Leibler JH, Sennett Decker CM, et al. High prevalence of chronic kidney disease of unknown etiology among workers in the Mesoamerican Nephropathy Occupational Study. BMC Nephrol. 2022;23(1):238. 35794550 10.1186/s12882-022-02861-0 PMC9261054

[r20] Climate Change Knowledge Porta. Guatemala Country Summary. Accessed July 1, 2024. https://climateknowledgeportal.worldbank.org/country/guatemala.

[r21] Watts N, Amann M, Arnell N, et al. The 2020 report of The Lancet Countdown on health and climate change: Responding to converging crises. Lancet. 2021;397(10269):129–170. 33278353 10.1016/S0140-6736(20)32290-X PMC7616803

[r22] National Institute for Occupational Safety and Health (NIOSH). What is Total Worker Health? 2020. Accessed January 18, 2023. https://www.cdc.gov/niosh/twh/totalhealth.html.

[r23] Damschroder LJ, Reardon CM, Widerquist MAO, Lowery J. The updated Consolidated Framework for Implementation Research based on user feedback. Implement Sci. 2022;17(1):75. 36309746 10.1186/s13012-022-01245-0 PMC9617234

[r24] Guerin RJ, Harden SM, Rabin BA, et al. Dissemination and implementation science approaches for occupational safety and health research: Implications for advancing Total Worker Health. Int J Environ Res Public Health. 2021;18(21):11050. 34769573 10.3390/ijerph182111050 PMC8583149

[r25] Burke WW, Litwin GH. A causal model of organizational performance and change. J Manage. 1992;18(3):523–545.

[r26] Schwatka NV, Tenney L, Newman LS. Protection and health promotion in small business. In: Burke R, Richardsen A, eds. Increasing Occupational Health and Safety in Workplaces: Research and Practice. Edward Elgar Publishing; 2019.

[r27] Burke MJ, Signal SM. Workplace safety: A multilevel interdisciplinary perspective. In: Liao H, Martocchio J, Joshi A, eds. Research in Personnel and Human Resources Management. Vol 29. Emerald Group Publishing Limited; 2010:1–47.

[r28] Eriksen M, Rothenberg R. Community-based participatory research (CBPR). Editorial. Health Educ Res. 2012;27(4):553–554. 22798615 10.1093/her/cys066

[r29] Jaramillo D, Krisher L, Schwatka NV, Tenney L. International Total Worker Health: Applicability to agribusiness in Latin America. Int J Environ Res Public Health. 2021;18(5):2252. 33668716 10.3390/ijerph18052252 PMC7956694

[r30] Guerin RJ, Glasgow RE, Tyler A, Rabin BA, Huebschmann AG. Methods to improve the translation of evidence-based interventions: A primer on dissemination and implementation science for occupational safety and health researchers and practitioners. Saf Sci. 2022;152:105763. 37854304 10.1016/j.ssci.2022.105763 PMC10583726

[r31] Glaser J, Hansson E, Weiss I, et al. Preventing kidney injury among sugarcane workers: Promising evidence from enhanced workplace interventions. Occup Environ Med. 2020;77(8):527–534. 32404530 10.1136/oemed-2020-106406 PMC7402461

[r32] Wegman DH, Apelqvist J, Bottai M, et al. Intervention to diminish dehydration and kidney damage among sugarcane workers. Scand J Work Environ Health. 2017;44(1):16–24. 28691728 10.5271/sjweh.3659

[r33] Bodin T, Garcia-Trabanino R, Weiss I, et al. Intervention to reduce heat stress and improve efficiency among sugarcane workers in El Salvador: Phase 1. Occup Environ Med. 2016;73(6):409–416. 27073211 10.1136/oemed-2016-103555 PMC4893112

[r34] Hansson E, Jakobsson K, Glaser JR, et al. Response to: Letter to the Editor-Productivity as an efficacy measure in rest-shade-hydration interventions: The need for a more complete dissemination and implementation science approach by Dally M and Newman LS. Ann Work Expo Health. 2024;68(6):668–671. 38829828 10.1093/annweh/wxae046

[r35] Lucas RAI, Hansson E, Skinner BD, et al. The work-recovery cycle of kidney strain and inflammation in sugarcane workers following repeat heat exposure at work and at home. Eur J Appl Physiol. 2025;125(3):639–652. 39369140 10.1007/s00421-024-05610-3 PMC11889006

[r36] Dally M, Newman LS. Productivity as an efficacy measure in rest-shade-hydration interventions: The need for a more complete dissemination and implementation science approach. Ann Work Expo Health. 2024;68(6):665–667. 38776124 10.1093/annweh/wxae044

[r37] Holkup PA, Tripp-Reimer T, Salois EM, Weinert C. Community-based participatory research: An approach to intervention research with a Native American community. ANS Adv Nurs Sci. 2004;27(3):162–175. 15455579 10.1097/00012272-200407000-00002 PMC2774214

[r38] Dally M, Krisher L, Macaluso F, James KA, Newman LS. Workers and climate change: The need for academic–industry partnerships to improve agricultural worker health, safety, and wellbeing. Sustainability. 2022;14(11):6717.

[r39] Glasgow RE, Harden SM, Gaglio B, et al. RE-AIM Planning and Evaluation Framework: Adapting to new science and practice with a 20-year review. Front Public Health. 2019;7:64. 30984733 10.3389/fpubh.2019.00064 PMC6450067

[r40] Dally M, Butler-Dawson J, Sorensen CJ, et al. Wet bulb globe temperature and recorded occupational injury rates among sugarcane harvesters in southwest Guatemala. Int J Environ Res Public Health. 2020;17(21):8195. 33171945 10.3390/ijerph17218195 PMC7664243

[r41] Butler-Dawson J, Krisher L, Yoder H, et al. Evaluation of heat stress and cumulative incidence of acute kidney injury in sugarcane workers in Guatemala. Int Arch Occup Environ Health. 2019;92(7):977–990. 30997573 10.1007/s00420-019-01426-3 PMC6768910

[r42] Sorensen CJ, Butler-Dawson J, Dally M, et al. Risk factors and mechanisms underlying cross-shift decline in kidney function in Guatemalan sugarcane workers. J Occup Environ Med. 2019;61(3):239–250. 30575695 10.1097/JOM.0000000000001529 PMC6416034

[r43] Krisher L, Butler-Dawson J, Yoder H, et al. Electrolyte beverage intake to promote hydration and maintain kidney function in Guatemalan sugarcane workers laboring in hot conditions. J Occup Environ Med. 2020;62(12):e696–e703. 33003044 10.1097/JOM.0000000000002033 PMC7720870

[r44] OSHA. OSHA Technical Manual Section III: Chapter 4 Heat Stress. 2017. Updated September 15, 2017. Accessed August 14, 2019. https://www.osha.gov/dts/osta/otm/otm_iii/otm_iii_4.html.

[r45] Sorensen CJ, Krisher L, Butler-Dawson J, et al. Workplace screening identifies clinically significant and potentially reversible kidney injury in heat-exposed sugarcane workers. Int J Environ Res Public Health. 2020;17(22):8552. 33218070 10.3390/ijerph17228552 PMC7698805

[r46] Schwatka NV, Brown CE, Tenney L, et al. Evaluation of a Total Worker Health® leadership program for small business leaders. Occup Health Sci. 2021;5:163–188. 37366387 10.1007/s41542-021-00086-5 PMC10291283

[r47] Schwatka NV, Dally M, Tenney L, Shore E, Brown CE, Newman LS. Total Worker Health leadership and business strategies are related to safety and health climates in small business. Int J Environ Res Public Health. 2020;17(6):2142. 32213806 10.3390/ijerph17062142 PMC7143812

[r48] Schwatka NV, Jaramillo D, Dally M, et al. Latin American agricultural workers’ job demands and resources and the association with health behaviors at work and overall health. Front Public Health. 2022;10:838417. 35462804 10.3389/fpubh.2022.838417 PMC9021611

[r49] Jaramillo D, Clancy RL, Fisher GG, et al. Evaluation of a Total Worker Health leadership training in Latin America: Opportunities for sustainable change in low- and middle-income countries. BMC Public Health. March 12, 2025;25(1):984. doi:10.1186/s12889-025-22179-4. 40075341 PMC11905678

[r50] Krisher L, Butler-Dawson J, Schlosser K, et al. Body composition, anemia, and kidney function among Guatemalan sugarcane workers. Nutrients. 2021;13(11):3928. 34836182 10.3390/nu13113928 PMC8621317

[r51] Krisher L. Recovery from work in the heat: Contributions of workday and off-work exposures on the health of Guatemala sugarcane workers. [Unpublished doctoral dissertation]. Colorado School of Public Health, University of Colorado; 2023.

[r52] Butler-Dawson J, Krisher L, Asensio C, et al. Risk factors for declines in kidney function in sugarcane workers in Guatemala. J Occup Environ Med. 2018;60(6):548–558. 29370016 10.1097/JOM.0000000000001284 PMC5991184

[r53] Dally M, Dye-Robinson A, Li Y, et al. Performance of summary measures of core body temperature in heat-related health outcome regression models. Int J Biometeorol. June 12, 2025. doi:10.1007/s00484-025-02957-w. Online ahead of print. 40504364

[r54] Adgate JL, Erlandson G, Butler-Dawson J, Butler-Dawson J. Airborne particulate matter exposure in male sugarcane workers at risk for chronic kidney disease in Guatemala. Ann Work Expo Health. 2025;69(377–388). 10.1093/annweh/wxaf008 PMC13368822 40072364

[r55] Stumpf AM, Erlandson G, Ruths J, et al. Noise exposures of sugar cane mill workers in Guatemala. Int J Audiol. 2020:59(sup1):S48–S53. 31738616 10.1080/14992027.2019.1690171 PMC12861511

[r56] Barrientos DM, Krisher L, Cruz A, et al. SARS-COV-2 seroprevalence, COVID-19 vaccination, and hesitancy in agricultural workers in rural Guatemala. American Society of Tropical Medicine & Hygiene Annual Meeting; October 30 - November 3, 2022. Seattle, WA, USA.

[r57] Tenney L, Huebschmann AG, Brown CE, Schwatka NV, Newman LS. Leveraging an implementation science framework to measure the impact of efforts to scale out a Total Worker Health® intervention to employers. Int J Environ Res Public Health. 2022;19(3):1372. 35162408 10.3390/ijerph19031372 PMC8834848

[r58] Milanesio J. La agroindustria azucarera latinoamericana trabaja para unir al sector en la región. 2024. Accessed August 12, 2024. https://newsweekespanol.com/2024/01/02/la-industria-azucarera-de-guatemala-trabaja-para-unir-al-sector-en-toda-latinoamerica/.

[r59] Dally M, Macaluso F, James KA, Newman LS, Sorensen CJ. Addressing climate change in the workplace. Workplace Health Saf. 2022;70(7):340. 35822465 10.1177/21650799221103767

